# Intracranial and extracranial artery stenosis and clinical outcome of acute ischemic stroke patients receiving intravenous thrombolysis

**DOI:** 10.3389/fneur.2025.1700753

**Published:** 2026-01-23

**Authors:** Yangyang Guo, Bingyang Zhang, Lianmei Zhong, Chunyan Lei

**Affiliations:** First Department of Neurology, The First Affiliated Hospital of Kunming Medical University, Kunming, Yunnan, China

**Keywords:** acute ischemic stroke, clinical outcome, hemorrhagic transformation, intracranial and extracranial artery stenosis, intravenous thrombolysis

## Abstract

**Background:**

Intracranial and/or extracranial atherosclerotic stenosis is a common etiology of acute ischemic stroke (AIS). This study aimed to evaluate the impact of intracranial or extracranial atherosclerotic stenosis on early neurological deterioration (END), hemorrhagic transformation (HT) and 90-day clinical outcomes in patients receiving intravenous thrombolysis.

**Methods:**

We retrospectively enrolled patients with AIS who received intravenous alteplase (0.9 mg/kg) at the First Affiliated Hospital of Kunming Medical University between February 2019 and August 2022. Data on demographics, stroke risk factors, laboratory results, and neuroimaging findings were collected. Atherosclerotic stenosis (AS) was defined as >50% intracranial or extracranial arteries. Logistic regression was performed to identify independent predictors of clinical outcomes. END was defined as an increase of ≥4 points in the National Institutes of Health Stroke Scale (NIHSS) score within 24 h after stroke onset. HT was defined as any newly detected intracranial hemorrhage on follow-up cranial CT performed within 7 days after symptom onset.

**Results:**

A total of 185 AIS patients receiving intravenous thrombolysis were included in this study, with 88 (47.6%) in the IEAS group and 97 (52.4%) in the non-stenosis group. There was no significant association between the incidence of END and the presence of IEAS. Multivariable regression analysis revealed that baseline NIHSS was an independent risk factor for HT (OR = 1.120, 95% CI 1.038–1.209, *p* = 0.003), 90-day poor clinical outcome (OR = 1.198, 95% CI 1.105–1.298, *p* = 0.001) and 90-day death (OR = 1.384, 95% CI 1.179–1.625, *p* = 0.001). Although IEAS was not significantly associated with the incidence of END or HT, it was significantly correlated with 90-day poor clinical outcome (OR = 1.350, 95% CI 1.108–1.644, *p* = 0.003).

**Conclusions:**

In this cohort, IEAS was not associated with END or HT but emerged as an independent predictor of poor 90-day functional outcome after intravenous thrombolysis for AIS.

## Introduction

Acute ischemic stroke (AIS) is a severe cerebrovascular disorder with a substantial global burden. Annually, it affects approximately 15 million individuals worldwide, leading to over 6 million deaths and more than 5 million cases of permanent disability (1). Epidemiological data indicate that the global mortality rate of ischemic stroke exceeds 10%, and two-thirds of the survivors are left with disabilities (2). Intravenous thrombolysis (IVT) is a cornerstone of acute treatment, significantly improving outcomes when administered within 4.5 h of symptom onset and is strongly endorsed by international guidelines (3–5).

In recent years, advances such as the widespread use of antiplatelet agents, carotid artery stenting, and novel therapeutic and neuroprotective strategies have contributed to a decline in AIS mortality. Nevertheless, only a subset of patients achieve fully independent neurological recovery (6). Previous studies suggest that AIS patients with coexisting extracranial atherosclerotic stenosis (ECAS) face a higher short-term risk of recurrence after IVT, which may be attributed to micro-thromboembolism from plaque or thrombus fragmentation (7, 8). Moreover, even following successful recanalization, neurological recovery in these patients may be hampered by persistent chronic hypoperfusion (9). Additionally, atherosclerotic stenosis has been linked to an elevated risk of hemorrhagic transformation (HT), particularly in the presence of comorbidities such as hypertension and diabetes mellitus (10).

Intracranial or extracranial atherosclerotic stenosis is present in approximately 31.0 to 62.3% of patients with acute ischemic stroke (AIS) (11). Intracranial atherosclerotic stenosis (ICAS) is particularly common, with one study of 1,006 AIS patients reporting an ICAS prevalence of 32.9%, of which 19.98% involved the anterior circulation and 7.95% the posterior circulation (12). Within the anterior circulation, middle cerebral artery stenosis is the most frequently observed (13), likely resulting from the interplay of genetic factors-such as ApoE and the PI3K-Akt signaling pathway (14, 15) and hemodynamic shear stress (16). Research indicated that ICAS was associated with early neurological deterioration (END) in patients receiving IVT (17), often attributed to hypoperfusion due to stenosis and failure of collateral circulation, leading to expansion of the ischemic penumbra (18). Especially when ICAS is located in the anterior circulation with 50–99% stenosis, compromised cerebral perfusion may predispose patients to neurological deterioration (19).

Previous studies have indicated that AS was an independent factor for END and 90-day adverse neurological outcomes in AIS patients. However, clinical studies examining the relationship between intracranial or extracranial atherosclerotic stenosis and clinical outcomes in AIS patients undergoing IVT are limited. In this study, we therefore aimed to examine the associations between the presence of intracranial or extracranial atherosclerotic stenosis and functional outcomes and hemorrhagic transformation (HT) in AIS patients treated with IVT.

## Methods and materials

This retrospective study included patients with acute ischemic stroke (AIS) who received intravenous thrombolysis (IVT) at the First Affiliated Hospital of Kunming Medical University between February 2019 and August 2022.

Inclusion criteria: (1) age ≥18 years; (2) Patients with acute ischemic stroke confirmed by cranial magnetic resonance imaging diffusion-weighted imaging (MRI-DWI); (3) provision of written informed consent for IVT by the patient or legal surrogate prior to treatment; (4) IVT administration within 4.5 h of symptom onset; (5) availability of routine cranial computed tomography (CT) and magnetic resonance imaging (MRI) performed before IVT and within 24 h after treatment.

Exclusion criteria included: (1) coexistence of other intracranial pathologies or severe systemic organ diseases (e.g., intracranial aneurysm, brain tumor); (2) pre-existing intracranial hemorrhage or documented bleeding tendency; (3) refusal of treatment by the family or inability to complete follow-up; (4) incomplete cranial MRI examination.

### Intravenous thrombolysis protocol

For eligible patients who provided informed consent and had no absolute contraindications, intravenous thrombolysis was performed using alteplase at a dose of 0.9 mg/kg. The agent was administered as an initial bolus (10% of the total dose) over 1 min, followed by infusion of the remaining 90% over 1 h. All enrolled patients received treatment within 4.5 h of symptom onset, consistent with current guideline recommendations. Extended time windows (e.g., up to 9 h based on perfusion imaging selection) were not applied in this study.

### Baseline data collection

The collected clinical data mainly includes: (1) demographic data: gender, age; (2) risk factors of cerebrovascular disease: smoking, drinking, hypertension, diabetes, history of stroke and history of coronary heart disease; (3) admission status: time from onset to hospital, baseline NIHSS score, systolic and diastolic blood pressure at admission; (4) blood biochemical indicators within 24 h: hemoglobin, platelet count; (5) imaging indicators: location and degree of intracranial and extracranial atherosclerotic stenosis, HT; (6) venous thrombolysis dose; (7) other: TOAST classification, location of cerebral infarction.

### Definition of END and HT

Early neurological deterioration (END) was defined as an increase of ≥4 points in the National Institutes of Health Stroke Scale (NIHSS) score within 24 h after stroke onset, after excluding other identifiable causes (20).

HT was defined as any newly detected intracranial hemorrhage on follow-up cranial CT performed within 7 days after symptom onset (21).

### Definition of subgroup

AS was defined as any atherosclerotic narrowing identified in the intracranial or extracranial arteries. ICAS referred to stenosis affecting the anterior cerebral artery, middle cerebral artery, posterior cerebral artery, intracranial segment of the internal carotid artery (C2 segment and above), basilar artery, or intracranial segment of the vertebral artery (V4 segment and above). ECAS was defined as stenosis of the internal carotid artery from the C1 segment or the extracranial vertebral artery below the V4 segment. Concomitant IEAS indicated the presence of both ICAS and ECAS in the same patient. This composite IEAS category was used in the primary analyses to represent advanced, diffuse atherosclerotic burden involving the entire cerebral arterial system. Patients with combined intracranial and extracranial stenosis may experience greater hemodynamic compromise and reduced collateral compensatory capacity, which could collectively affect clinical outcomes following IVT.

### Diagnosis of intracranial and extracranial atherosclerotic stenosis

Magnetic resonance imaging (MRI) was performed on 3.0 T scanners (Siemens AG, Germany, Philips). The imaging protocol included T1-weighted imaging (T1WI), T2-weighted imaging (T2WI), fluid-attenuated inversion recovery (FLAIR), and magnetic resonance angiography (MRA). The intracranial arteries include the anterior cerebral artery, middle cerebral artery, posterior cerebral artery, and internal carotid artery.

Arterial stenosis was independently evaluated by two experienced radiologists who were blinded to the patient’s clinical information. In cases of disagreement between the two radiologists, the evaluation was conducted by a third radiologist or a higher-level physician. The calculation of ICAS was based on the Warfarin-Aspirin Symptomatic Intracranial Disease Trial, with the formula as follows: Stenosis rate = 1 − (diameter of the narrowest part of the diseased vessel/diameter of the normal proximal part of the diseased vessel). The calculation of ECAS was based on the North American Symptomatic Carotid Endarterectomy Trial, with the formula as follows: (diameter of the normal site at the distal end of the stenosis - diameter of the narrowest site of the diseased vessel)/diameter of the normal site at the distal end of the stenosis.

Definitions: Mild stenosis: 1 to 49%; Moderate stenosis: 50 to 69%; Severe stenosis: 70 to 99%; Occlusion: >99% occlusion. According to the calculation results, any intracranial or extracranial atherosclerotic stenosis rate >50% was defined as the atherosclerotic stenosis group (22).

### Statistical analysis

All statistical analyses and data plotting were performed using SPSS and GraphPad Prism. Normality tests were conducted on continuous data. If the continuous data follow a normal distribution, they were expressed as mean ± standard deviation (x ± s). Independent two-sample t-tests were used for intergroup comparisons. Non-normally distributed data are expressed as median (interquartile range, IQR) and were compared with the Mann–Whitney *U* test. Categorical data were expressed as the number of cases (percentage), with intergroup comparisons performed using the Chi-squared test, continuity correction, or Fisher’s exact test. Variables identified as significant by univariate analyses (*p* < 0.05) or considered clinically relevant were included in multivariate logistic regression analysis to determine the association between intracranial or extracranial atherosclerotic stenosis and clinical outcomes.

## Results

### Overall characteristics

**Figure 1:**
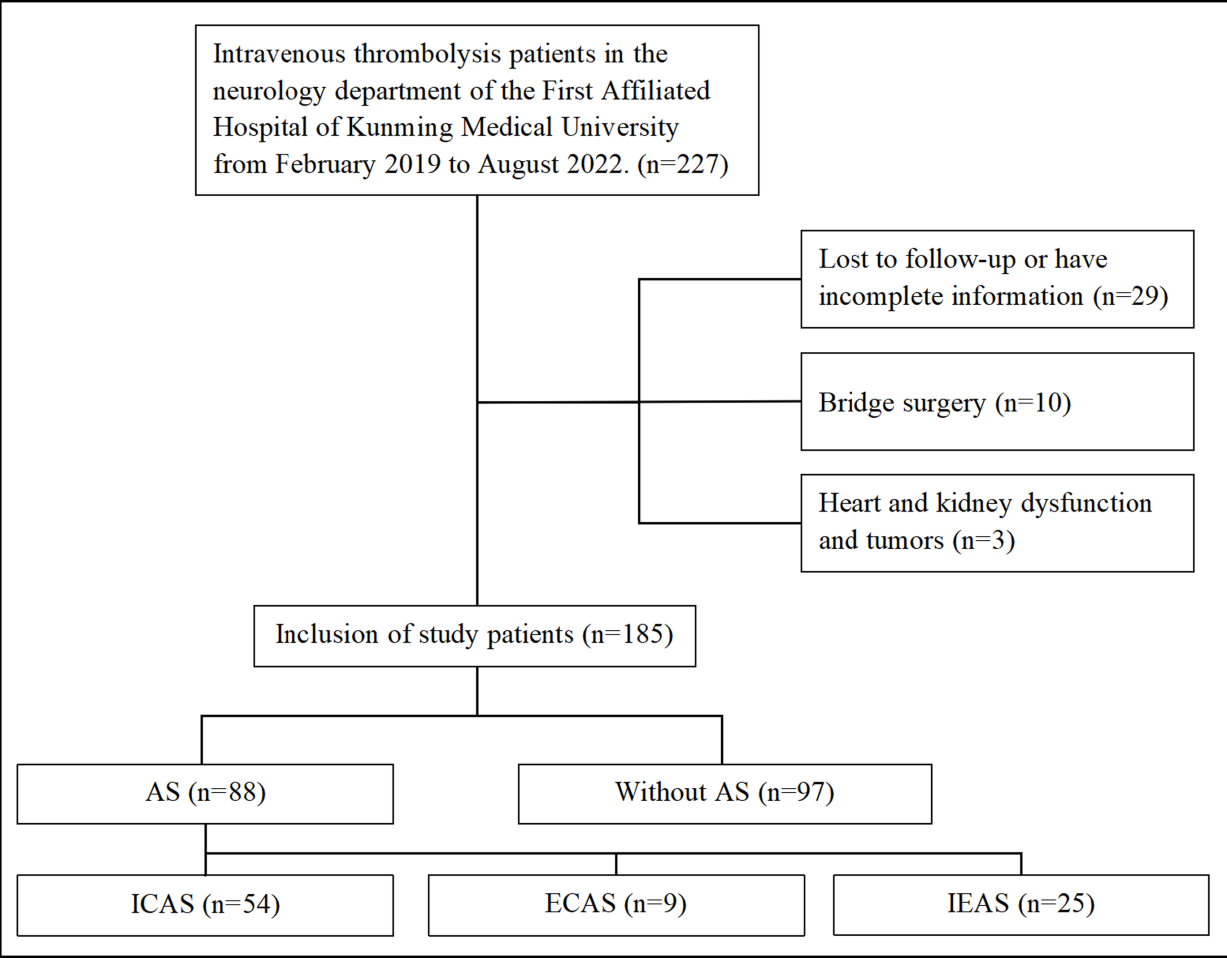
Flow chart of research object enrollment. AS, Atherosclerotic stenosis; ICAS, Intracranial atherosclerotic stenosis; ECAS, Extracranial atherosclerotic stenosis; IEAS, Intracranial and extracranial atherosclerotic stenosis.

A total of 227 AIS patients who received IVT within 4.5 h of symptom onset were enrolled. Of these, 10 patients were excluded due to bridging surgery; 10 were excluded due to missing information; 3 were excluded due to severe cardiac and renal dysfunction, and tumors; 19 patients were lost to follow-up at 3 months. Ultimately, 185 patients were included in the study. Of these, 88 (47.6%) cases were in the AS group, and 97 (52.4%) cases were in the group without AS (Figure 1). Among all patients with intracranial and extracranial atherosclerotic stenosis, 54 (29.2%) cases had ICAS, 9 (4.9%) cases had ECAS, and 25 (13.5%) cases had IEAS. Based on the degree of stenosis classification, 18 (9.7%) cases had moderate stenosis, 14 (7.6%) cases had severe stenosis, 30 (16.2%) cases had arterial occlusion, and 26 (14.1%) cases had mixed stenosis (Table 1).

**Table 1:** Location and severity of atherosclerotic stenosis.

Variables	All patients (*n* = 185)	Without AS (*n* = 97)	ICAS (*n* = 54)	ECAS (*n* = 9)	IEAS (*n* = 25)
No-mild stenosis	97 (54.4)	97 (100)	0 (0)	0 (0)	0 (0)
Moderate stenosis	18 (9.7)	0 (0)	15 (27.8)	3 (33.3)	0 (0)
Severe stenosis	14 (7.6)	0 (0)	6 (11.1)	4 (44.4)	4 (16.0)
artery occlusion	30 (16.2)	0 (0)	20 (37.0)	1 (11.1)	9 (36.0)
Mixed stenosis	26 (14.1)	0 (0)	13 (24.1)	1 (11.1)	12 (48.0)

AS, Atherosclerotic stenosis; ICAS, Intracranial atherosclerotic stenosis; ECAS, Extracranial atherosclerotic stenosis; IEAS, Intracranial and extracranial atherosclerotic stenosis.

### Baseline characteristics of patients with AIS with or without END

There were no statistically significant differences in age, gender, hypertension, diabetes, biochemical indicators, and other examinations (Table 2). Among all patients, 19 (10.2%) cases experienced END. The demographic and clinical data of patients in the END and without END groups were not statistically significant (Table 3). Furthermore, the rate of AS did not differ significantly between the END and without END groups.

**Table 2:** The baseline data and clinical data between atherosclerotic stenosis group and without atherosclerotic stenosis group.

Variables	All patients (*n* = 185)	AS (*n* = 88)	Without AS (*n* = 97)	*p* value
Demographic				
Age (years, Mean ± SD)	65.70 ± 13.95	67.67 ± 12.72	63.91 ± 14.80	0.067
Male (*n*, %)	112 (60.5)	51 (58.0)	61 (62.9)	0.493
Vascular risk factor (*n*, %)				
Hypertension	125 (67.6)	62 (70.5)	63 (64.9)	0.424
Diabetes	46 (24.9)	27 (30.7)	19 (19.6)	0.081
Coronary heart disease	13 (7.0)	6 (6.8)	7 (7.2)	0.916
Atrial fibrillation	20 (10.8)	7 (8.0)	13 (14.4)	0.098
Prior stroke	29 (15.7)	15 (17.0)	14 (14.4)	0.625
Smoking	59 (31.9)	27 (30.7)	32 (33.0)	0.737
Drinking	25 (13.5)	10 (11.4)	15 (15.5)	0.415
BMI (kg/m^2^, Mean ± SD)	23.56 ± 3.21	23.68 ± 3.28	23.45 ± 3.17	0.634
Baseline NIHSS [Median (IQR)]	5 (3, 9)	6 (3, 9)	4 (3, 7)	0.063
ASBP (mmHg, Mean ± SD)	143.92 ± 24.03	141.36 ± 25.90	146.25 ± 22.08	0.168
ADBP (mmHg, Mean ± SD)	81.68 ± 14.10	79.60 ± 14.68	83.57 ± 13.35	0.056
Laboratory examination				
PLT [10^9^/L, Median (IQR)]	200.0 (162.5, 239.5)	200.0 (160.3, 247.5)	203.0 (163.5, 237.5)	0.796
FBG [mmol/L, Median (IQR)]	5.4 (4.7, 6.9)	5.9 (4.8, 7.3)	5.3 (4.6, 6.5)	0.058
APTT (s, Mean ± SD)	37.69 ± 5.28	38.47 ± 5.14	36.93 ± 5.35	0.064
Location of cerebral infarction (*n*, %)				0.907
Anterior circulation	152 (82.5)	72 (81.8)	80 (82.5)	
Posterior circulation	33 (17.8)	16 (18.2)	17 (17.5)	

AS, Atherosclerotic stenosis; NIHSS, National Institutes of Health Stroke Scale; BMI, Body Mass Index; ASBP, Admission systolic blood pressure; ADBP, Admission diastolic blood pressure; PLT, Platelet; FBG, Fasting blood glucose; APTT, Activated partial thromboplastin time.

**Table 3:** The baseline data and clinical data between early neurological deterioration group and without early neurological deterioration group.

Variables	All patients (*n* = 185)	END (*n* = 19)	With END (*n* = 166)	*p* value
Demographic				
Age (years, Mean ± SD)	65.70 ± 13.95	66.47 ± 13.36	65.61 ± 14.05	0.800
Male (*n*, %)	112 (60.5)	10 (52.6)	102 (61.4)	0.457
Vascular risk factors (*n*, %)				
Hypertension	125 (67.6)	13 (68.4)	112 (67.5)	0.933
Diabetes	46 (24.9)	4 (21.1)	42 (25.3)	0.900
Coronary heart disease	13 (7.0)	1 (5.3)	12 (7.2)	1.000
Atrial fibrillation	20 (10.8)	3 (15.8)	17 (10.2)	0.461
Prior stroke	29 (15.7)	2 (10.5)	27 (16.3)	0.750
Smoking	59 (31.9)	6 (31.6)	53 (31.9)	0.975
Drinking	25 (13.5)	3 (15.8)	22 (13.3)	1.000
BMI (kg/m^2^, Mean ± SD)	23.56 ± 3.21	23.52 ± 3.49	23.56 ± 3.19	0.956
Baseline NIHSS [Median (IQR)]	5 (3, 9)	6 (4, 9)	5 (2, 9)	0.200
ASBP (mmHg, Mean ± SD)	143.92 ± 24.03	149.36 ± 23.06	143.30 ± 24.12	0.298
ADBP (mmHg, Mean ± SD)	81.68 ± 14.10	84.89 ± 13.74	81.31 ± 14.13	0.296
Laboratory examination				
PLT [10^9^/L, Median (IQR)]	200.0 (162.5, 239.5)	218.0 (159, 249)	194.5 (162.8, 238)	0.339
FBG [mmol/L, Median (IQR)]	5.4 (4.7, 6.9)	5.9 (5.3, 7.9)	5.3 (4.7, 6.9)	0.151
APTT (s, Mean ± SD)	37.69 ± 5.28	37.82 ± 5.44	36.55 ± 3.48	0.324
Location of cerebral infarction				0.944
Anterior circulation (*n*, %)	152 (82.2)	15 (78.9)	137 (82.5)	
Posterior circulation (*n*, %)	33 (17.8)	2 (21.1)	29 (17.5)	
Atherosclerotic stenosis (*n*, %)				0.615
Without AS	97 (52.4)	11 (57.9)	86 (51.8)	
ICAS	54 (29.2)	4 (21.1)	50 (30.1)	
ECAS	9 (4.9)	1 (5.3)	8 (4.8)	
IEAS	25 (13.5)	3 (15.8)	22 (13.3)	

END, Early neurological deterioration; BMI, Body Mass Index; NIHSS, National Institutes of Health Stroke Scale; ASBP, Admission systolic blood pressure; ADBP, Admission diastolic blood pressure; PLT, Platelet; FBG, Fasting blood glucose; APTT, Activated partial thromboplastin time; AS, Atherosclerotic stenosis; ICAS, Intracranial atherosclerotic stenosis; ECAS, Extracranial atherosclerotic stenosis; IEAS, Intracranial and extracranial atherosclerotic stenosis.

### Baseline characteristics of patients with AIS with or without HT

A total of 19 (10.3%) cases experienced HT among all AIS patients who received IVT. Patients with HT had higher baseline NIHSS scores [12 (9.17) vs. 4 (2.8), *p* < 0.001] and a higher proportion of a smoking history [2 (10.5) vs. 57 (35.3), *p* = 0.035]. The rate of AS did not significantly differ between the HT and without HT groups (Table 4).

**Table 4:** The baseline data and clinical data between hemorrhagic transformation group and without hemorrhagic transformation group.

Variables	All patients (*n* = 185)	HT (*n* = 19)	Without HT (*n* = 166)	*p* value
Demographic				
Age (years, Mean ± SD)	65.70 ± 13.95	70.79 ± 14.12	65.12 ± 13.85	0.093
Male (*n*, %)	112 (60.5)	8 (42.1)	104 (62.7)	0.083
Vascular risk factors (*n*, %)				
Hypertension	125 (67.6)	13 (68.4)	112 (67.5)	0.933
Diabetes	46 (24.9)	4 (21.1)	42 (25.3)	0.900
Coronary heart disease	13 (7.0)	2 (10.5)	11 (6.6)	0.876
Atrial fibrillation	20 (10.8)	4 (21.1)	16 (9.6)	0.129
Prior stroke	29 (15.7)	5 (26.3)	24 (14.5)	0.311
Smoking	59 (31.9)	2 (10.5)	57 (34.3)	0.035*
Drinking	25 (13.5)	1 (5.3)	24 (14.5)	0.449
BMI (kg/m^2^, Mean ± SD)	23.56 ± 3.21	23.09 ± 3.80	23.61 ± 3.15	0.503
Baseline NIHSS (Median (IQR))	5 (3, 9)	12 (9, 17)	4 (2, 8)	<0.001*
ASBP (mmHg, Mean ± SD)	143.92 ± 24.03	142.58 ± 26.46	144.08 ± 23.81	0.797
ADBP (mmHg, Mean ± SD)	81.68 ± 14.10	81.37 ± 13.81	81.72 ± 14.18	0.919
Laboratory examination				
PLT (10^9^/L, Median (IQR))	37.69 ± 5.28	37.78 ± 5.29	36.92 ± 5.26	0.502
FBG (mmol/L, Median (IQR))	200 (162.5, 239.5)	202 (152, 249)	200 (162.8, 238.8)	0.849
APTT (s, Mean ± SD)	5.4 (4.7, 6.9)	6 (5.3, 6.8)	5.3 (4.7, 6.9)	0.135
Location of cerebral infarction				0.574
Anterior circulation (*n*, %)	152 (82.2)	17 (89.5)	135 (81.3)	
Posterior circulation (*n*, %)	33 (17.8)	2 (10.5)	31 (18.7)	
Atherosclerotic stenosis (*n*, %)				0.055
Without AS	97 (52.4)	6 (31.6)	91 (54.9)	
ICAS	54 (29.2)	8 (42.1)	46 (27.7)	
ECAS	9 (4.9)	1 (5.3)	8 (4.8)	
IEAS	25 (13.5)	4 (21.1)	21 (12.7)	

HT, Hemorrhagic transformation; BMI, Body Mass Index; NIHSS, National Institutes of Health Stroke Scale; ASBP, Admission systolic blood pressure; ADBP, Admission diastolic blood pressure; PLT, Platelet; FBG, Fasting blood glucose; APTT, Activated partial thromboplastin time; AS, Atherosclerotic stenosis; ICAS, Intracranial atherosclerotic stenosis; ECAS, Extracranial atherosclerotic stenosis; IEAS, Intracranial and extracranial atherosclerotic stenosis. *p < 0.05.

### Multivariable logistic regression analysis of HT

**Figure 2:**
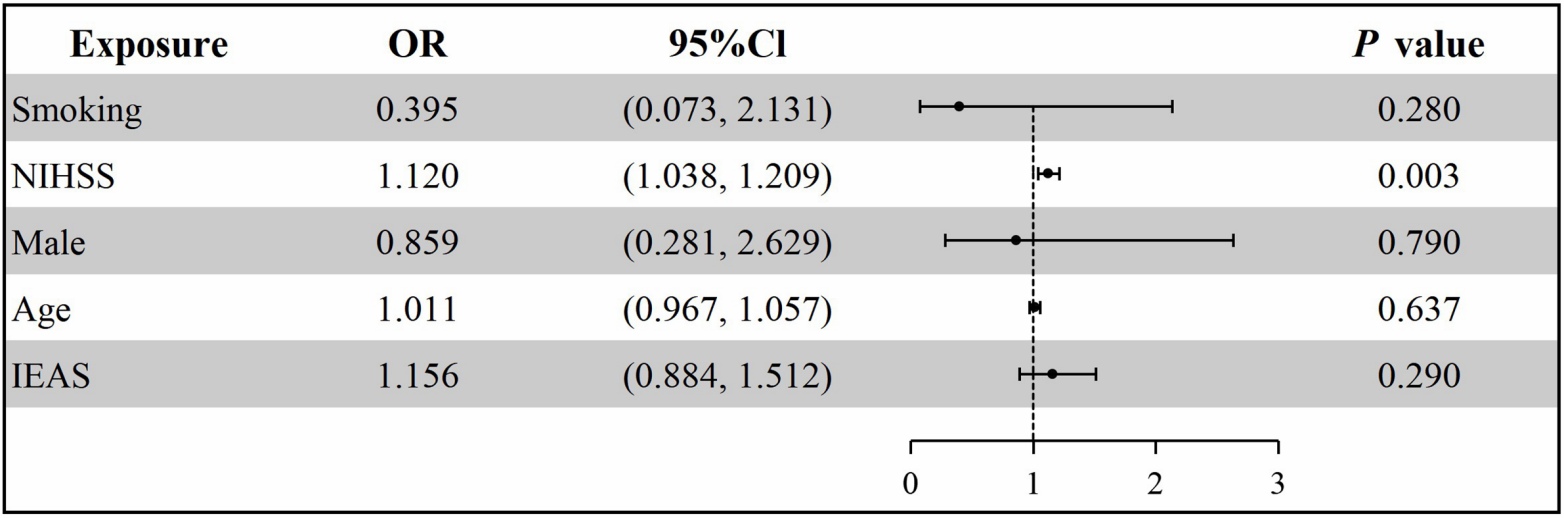
Multivariable logistic regression analysis for hemorrhagic transformation. NIHSS, National Institutes of Health Stroke Scale; IEAS, intracranial and extracranial atherosclerotic stenosis.

Variables with *p* < 0.05 from the univariate analysis, including gender, smoking history, age, baseline NIHSS score, and IEAS, were included in the multivariable regression analysis model. Multivariate logistic regression analysis indicated that a higher baseline NIHSS score was an independent risk factor for HT (OR = 1.120, 95% CI: 1.038–1.209, *p* = 0.003) in patients following IVT (Figure 2; Supplementary Table S1).

### The clinical characteristics between different clinical outcome groups

According to the 90-day mRS score, 139 (73.5%) patients had a good clinical outcome (mRS 0–2), and 49 (26.5%) patients had a poor clinical outcome (mRS 3–6). Patients in the poor clinical outcome group were older [(70.57 ± 12.90) vs. (63.95 ± 13.93), *p* = 0.004] and had higher baseline NIHSS scores [9 (5, 15.5) vs. 4 (2, 7), *p* < 0.001]. Patients in the poor clinical outcome group had prolonged activated partial thromboplastin time [38.98 ± 6.28 vs. 37.22 ± 4.81, *p* = 0.045], higher fasting blood glucose levels [5.9 (5.2, 7.0) vs. 5.3 (4.6, 6.9), *p* = 0.012] and higher rate of IEAS (Table 5).

**Table 5:** The baseline data and clinical data between good clinical outcome group and poor clinical outcome group.

Variables	All patients (*n* = 185)	Good clinical outcome (*n* = 136)	Poor clinical outcome (*n* = 49)	*p* value
Demographic				
Age (years, Mean ± SD)	65.70 ± 13.95	63.95 ± 13.93	70.57 ± 12.90	0.004*
Male (*n*, %)	112 (60.5)	83 (61.0)	29 (59.2)	0.821
Vascular risk factors (*n*, %)				
Hypertension	125 (67.6)	90 (66.2)	35 (71.4)	0.501
Diabetes	46 (24.9)	35 (25.7)	11 (22.4)	0.648
Coronary heart disease	13 (7.0)	10 (7.4)	3 (6.1)	1.000
Atrial fibrillation	29 (15.7)	21 (15.4)	8 (16.3)	0.884
Prior stroke	20 (10.8)	11 (8.1)	9 (18.4)	0.461
Smoking	59 (31.9)	42 (30.9)	17 (34.7)	0.624
Drinking	25 (13.5)	18 (13.2)	7 (14.3)	0.854
BMI (kg/m^2^, Mean ± SD)	23.56 ± 3.21	23.32 ± 3.10	24.21 ± 3.45	0.099
Baseline NIHSS (Median (IQR))	5 (3, 9)	4 (2, 7)	9 (5, 15.5)	<0.001*
ASBP (mmHg, Mean ± SD)	143.92 ± 24.03	144.79 ± 25.24	141.53 ± 20.33	0.940
ADBP (mmHg, Mean ± SD)	81.68 ± 14.10	81.73 ± 14.51	81.55 ± 13.01	0.236
Laboratory examination				
PLT [10^9^/L, Median (IQR)]	200.0 (162.5, 239.5)	194.5 (164.5, 236.8)	204.0 (152.5, 249.5)	0.940
FBG [mmol/L, Median (IQR)]	5.4 (4.7, 6.9)	5.3 (4.6, 6.9)	5.9 (5.2, 7.0)	0.012*
APTT (s, Mean ± SD)	37.69 ± 5.28	37.22 ± 4.81	38.98 ± 6.28	0.045*
Location of cerebral infarction				0.449
Anterior circulation (*n*, %)	152 (82.2)	110 (80.9)	42 (85.7)	
Posterior circulation (*n*, %)	33 (17.8)	26 (19.1)	7 (14.3)	
Atherosclerotic stenosis (*n*, %)				0.001*
Without AS	97 (52.4)	81 (59.6)	16 (32.7)	
ICAS	54 (29.2)	35 (25.7)	19 (38.8)	
ECAS	9 (4.9)	8 (5.9)	1 (2.0)	
IEAS	25 (13.5)	12 (8.8)	13 (26.5)	

BMI, Body Mass Index; NIHSS, National Institutes of Health Stroke Scale; ASBP, Admission systolic blood pressure; ADBP, Admission diastolic blood pressure; PLT, Platelet; FBG, Fasting blood glucose; APTT, Activated partial thromboplastin time; AS, Atherosclerotic stenosis; ICAS, Intracranial atherosclerotic stenosis; ECAS, Extracranial atherosclerotic stenosis; IEAS, Intracranial and extracranial atherosclerotic stenosis. *p < 0.05.

Among all patients, 13 (7.0%) died within 90 days. In the death group, baseline NIHSS scores were higher [18 (14, 23) vs. 4 (2.3, 8); *p* < 0.001], whereas fasting blood glucose levels [6.8 (6.1, 8.0) vs. 5.3 (4.7, 6.9), *p* = 0.011] and creatinine levels [91.3 (77.3, 117.7) vs. 77.3 (67.2, 89.7), *p* = 0.015] were also higher in the death group than in the survival group (Table 6).

**Table 6:** The baseline data and clinical data between survival group and death group.

Variables	All patients (*n* = 185)	Survival (*n* = 172)	Death (*n* = 13)	*p* value
Demographic				
Age (years, Mean ± SD)	65.70 ± 13.95	65.17 ± 13.82	72.77 ± 14.16	0.058
Male (*n*, %)	112 (60.5)	104 (60.5)	8 (61.5)	0.939
Vascular risk factors (*n*, %)				
Hypertension	125 (67.6)	117 (68.0)	8 (61.5)	0.862
Diabetes	46 (24.9)	44 (25.6)	2 (15.4)	0.626
Coronary heart disease	13 (7.0)	10 (5.8)	3 (23.1)	0.101
Atrial fibrillation	20 (10.8)	17 (9.9)	3 (17.8)	0.461
Prior stroke	29 (15.7)	26 (15.1)	3 (23.1)	0.715
Smoking	59 (31.9)	57 (33.1)	2 (15.4)	0.185
Drinking	25 (13.5)	25 (14.5)	0 (0)	0.290
BMI (kg/m^2^, Mean ± SD)	23.56 ± 3.21	23.47 ± 3.17	24.66 ± 3.75	0.198
Baseline NIHSS (Median (IQR))	5 (3, 9)	4 (2.3, 8)	18 (14, 23)	<0.001*
ASBP (mmHg, Mean ± SD)	143.92 ± 24.03	143.30 ± 24.05	152.15 ± 23.00	0.201
ADBP (mmHg, Mean ± SD)	81.68 ± 14.10	81.49 ± 14.14	84.15 ± 13.85	0.513
Laboratory examination				
PLT [10^9^/L, Median (IQR)]	200.0 (162.5, 239.5)	191.5 (161.3, 236.8)	248 (105.5, 259)	0.053
FBG [mmol/L, Median (IQR)]	5.4 (4.7, 6.9)	5.3 (4.7, 6.9)	6.8 (6.1, 8.0)	0.011*
APTT (s, Mean ± SD)	37.69 ± 5.28	37.75 ± 5.40	36.91 ± 3.36	0.586
Location of cerebral infarction				1.000
Anterior circulation (*n*, %)	152 (82.2)	141 (82.0)	11 (84.6)	
Posterior circulation (*n*, %)	33 (17.8)	31 (18.0)	2 (15.4)	
Atherosclerotic stenosis (*n*, %)				0.028*
Without AS	97 (52.4)	94 (54.7)	3 (23.1)	
ICAS	54 (29.2)	48 (27.9)	6 (46.2)	
ECAS	9 (4.9)	9 (5.2)	0 (0)	
IEAS	25 (13.5)	21 (12.21)	4 (30.8)	

BMI, Body Mass Index; NIHSS, National Institutes of Health Stroke Scale; ASBP, Admission systolic blood pressure; ADBP, Admission diastolic blood pressure; PLT, Platelet; FBG, Fasting blood glucose; APTT, Activated partial thromboplastin time; AS, Atherosclerotic stenosis; ICAS, Intracranial atherosclerotic stenosis; ECAS, Extracranial atherosclerotic stenosis; IEAS, Intracranial and extracranial atherosclerotic stenosis. *p < 0.05.

### Multivariable logistic regression analysis of 90-day clinical outcomes

**Figure 3:**
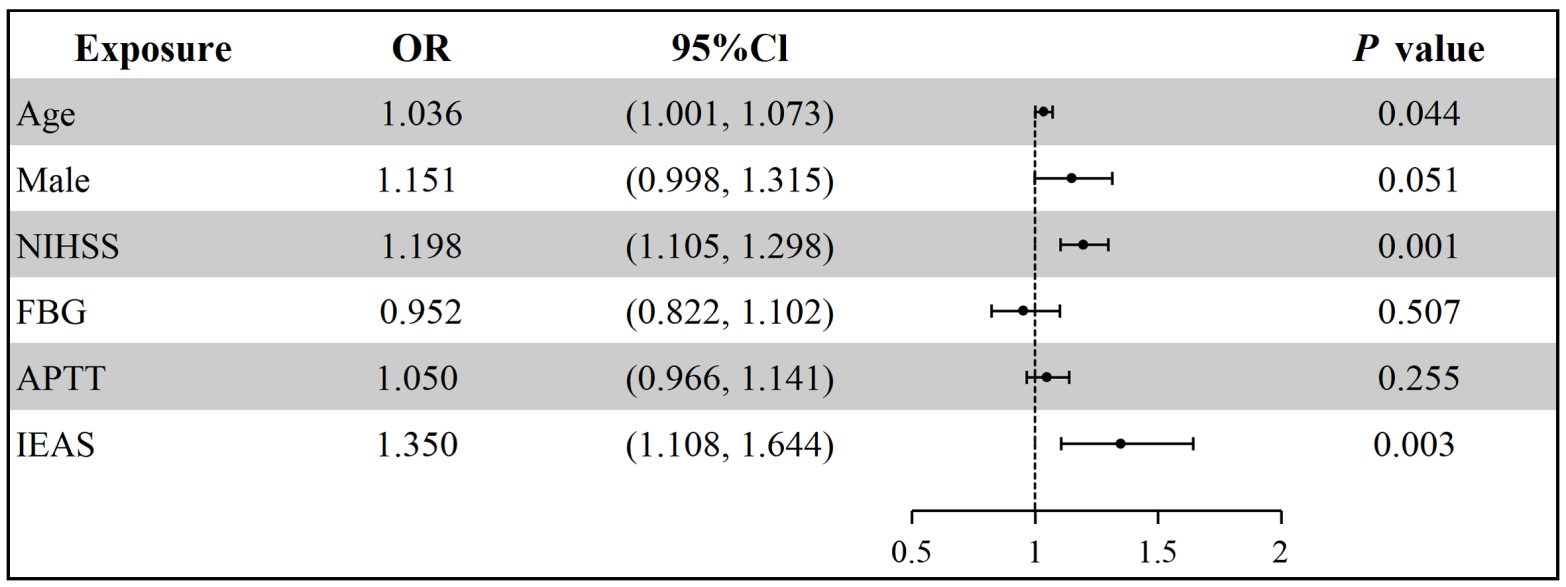
Multivariable logistic regression analysis for 90-day poor clinical outcome. NIHSS, National Institutes of Health Stroke Scale; FBG, Fasting blood glucose; APTT, Activated partial thromboplastin time; IEAS, intracranial and extracranial atherosclerotic stenosis.

**Figure 4:**
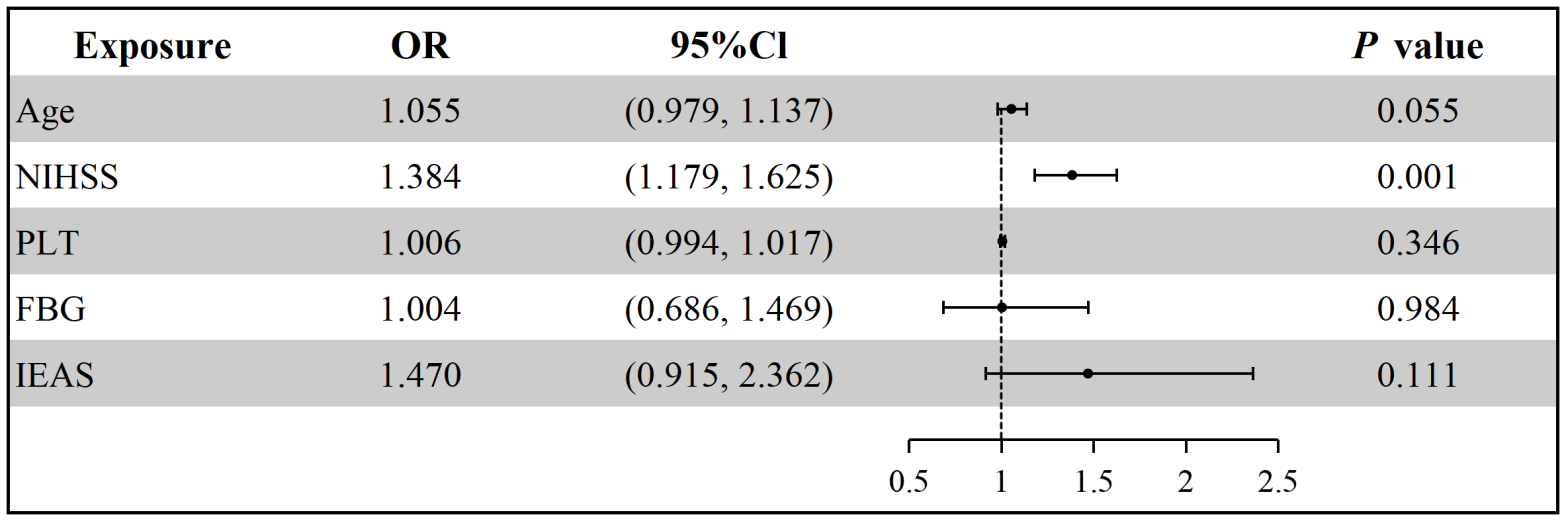
Multivariable logistic regression analysis for 90-day death. NIHSS, National Institutes of Health Stroke Scale; PLT, Platelet; FBG, Fasting blood glucose; IEAS, Intracranial and extracranial atherosclerotic stenosis.

Older patients (OR = 1.036, 95% CI: 1.001–1.073, *p* = 0.044) and those with IEAS (OR = 1.350, 95% CI: 1.108–1.644, *p* = 0.003) were more likely to experience a poor clinical outcome at 90 days (Figure 3; Supplementary Table S2). Additionally, the baseline NIHSS score (OR = 1.198, 95% CI: 1.105–1.298, *p* = 0.001) was an independent risk factor for poor clinical outcomes and increased the risk of death at 90 days (OR = 1.384, 95% CI: 1.179–1.625, *p* = 0.001) (Figure 4; Supplementary Table S3).

## Discussion

Our study compared the clinical baseline data, image features, treatment, and clinical outcome of AIS patients with and without AS. This study focused on the early and late indicators following IVT in patients with AS. The results suggested that IEAS was not an independent factor for END and HT, or 90-days mortality in patients undergoing IVT, but significantly increased the incidence of poor clinical outcomes at 90 days.

Although intracranial and extracranial atherosclerotic stenosis are anatomically distinct entities with different pathophysiological mechanisms, the coexistence of both lesions may reflect a more advanced stage of systemic large-artery atherosclerosis. Patients with IEAS were likely to experience compounded effects of proximal flow limitation, distal hypoperfusion, and reduced collateral capacity, which together may predispose them to poorer functional recovery despite successful intravenous thrombolysis. Therefore, IEAS was treated as a unified exposure variable in the primary analysis, aiming to capture the cumulative prognostic impact of diffuse arterial involvement rather than isolated anatomical lesions. In addition, in our study, the prevalence of isolated intracranial arterial stenosis was higher than that of isolated extracranial arterial stenosis, which was inconsistent with most previous reports and may be attributable to differences in study population characteristics, ethnic background, imaging assessment methods, or inclusion criteria (23).

Univariate analysis demonstrated a significant association between IEAS and 90-day mortality, however, this association was no longer significant after adjustment for baseline NIHSS score in the multivariable model. Previous studies have consistently shown that baseline NIHSS score was a well-established predictor of mortality after AIS, reflecting the initial severity of stroke (24, 25). Patients with concomitant AS often presented with more severe neurological deficits at admission and consequently higher baseline NIHSS scores, which were widely recognized as robust predictors of both short-term and long-term mortality following acute ischemic stroke. After inclusion of baseline NIHSS score in the multivariable model, it emerged as the sole independent predictor of 90-day mortality, whereas the effect of IEAS was attenuated. These findings suggested that IEAS may influence mortality indirectly by exacerbating initial stroke severity rather than exerting an independent and direct effect on death.

The reported incidence of END following AIS varied widely, ranging from 5 to 40% across studies (26). A study involving 306 patients with ischemic stroke treated with intravenous thrombolysis showed that approximately 10% of patients developed END (27). In our cohort, END occurred in 19 patients (10.2%), a rate consistent with previously reported figures. Although prior studies have identified AS or arterial occlusion as an independent risk factor for END in AIS patients (28, 29), our analysis did not demonstrate a significant association between AS and END. This discrepancy may be largely attributed to methodological variations in both the definition of END and the assessment of atherosclerotic stenosis. Earlier studies have employed heterogeneous definitions of END-often as an increase in NIHSS score of ≥2 or ≥4 points within 24–72 h (or up to 7 days) after stroke onset-and may have included deterioration due to secondary factors such as blood-pressure fluctuations, metabolic disturbances, or progressive cerebral edema (30–32). In contrast, we applied a more stringent criterion: an increase of ≥4 NIHSS points within 24 h of symptom onset after excluding other identifiable causes. While this approach likely reduced misclassification, it may also have lowered the overall incidence of END.

Furthermore, previous reports frequently focused on severe (≥70%) stenosis or complete occlusion of the culprit artery, typically evaluated with higher-resolution modalities such as CT angiography or digital subtraction angiography, which better delineated luminal narrowing and hemodynamic impairment (33, 34). In our study, arterial stenosis was assessed by MRA and defined as >50% narrowing in any intracranial or extracranial atherosclerotic segment, thereby encompassing a spectrum that included moderate stenosis, severe stenosis, and occlusion. Patients with moderate stenosis may retain sufficient collateral perfusion, potentially mitigating the effect of AS on early neurological decline (35). Additionally, our analysis did not separately evaluate stenosis in culprit versus non-culprit arteries, which could have further diluted any observable association between AS and END. Collectively, differences in END definitions, imaging techniques, and thresholds for grading stenosis severity likely contributed to the inconsistent findings across studies regarding the relationship between atherosclerotic stenosis and END.

In the landmark ECASS III trial, the incidence of HT was reported to be 27% (36). In our cohort of 185 patients, HT occurred in 19 cases, accounting for 10.3% of the total-a rate consistent with previous observations. Several predictive models for HT have been developed, including the HAT (37), SEDAN (38), SITS-SICH (39), and GRASPS (40) scores. However, all these models were derived from European populations; thus, their applicability to Asian cohorts requires further validation. Some studies have suggested that vascular calcification on the affected side was associated with HT after IVT (41, 42). Regarding age as a potential risk factor, consensus was lacking. One study of 126 patients found no increased risk of HT in individuals aged ≥80 years compared with those under 80 (43). In contrast, our analysis identified older age as a significant predictor of poor clinical outcome, with each additional year increasing the risk of an unfavorable prognosis by a factor of 1.039.

This study has several limitations. First, it was a single center investigation with a relatively small sample size, and most participants resided in high-altitude regions, which may limit the generalizability of the findings to other populations. Second, intracranial and extracranial atherosclerotic stenosis were assessed MRA. MRA was susceptible to signal loss due to turbulent flow, which can overestimate stenosis severity, and was prone to motion artifacts that may lead to misclassification of severe stenosis as complete occlusion (44, 45). Future studies should aim to recruit larger, multi-center cohorts to minimize regional and ethnic biases. Moreover, employing more precise vascular imaging techniques (e.g., high-resolution MRI, CT angiography, or digital subtraction angiography) would allow for a more accurate evaluation of stenosis and its hemodynamic impact. Such approaches will help clarify the early and long-term effects of intracranial and extracranial atherosclerotic stenosis on clinical outcomes after IVT for AIS, ultimately providing stronger evidence to guide clinical management.

IEAS and advanced age were identified as independent risk factors for poor 90-day clinical outcomes in patients receiving intravenous thrombolysis. In addition, a higher baseline NIHSS score was independently associated with hemorrhagic transformation and unfavorable long-term clinical outcomes in acute ischemic stroke patients.

## Data Availability

The raw data supporting the conclusions of this article will be made available by the authors, without undue reservation.
