# Supplementary material for: Intracranial and extracranial artery stenosis and clinical outcome of acute ischemic stroke patients receiving intravenous thrombolysis
Source: Front Neurol. 2026 Jan 23;16:1700753. doi: 10.3389/fneur.2025.1700753 (PMC12878150; doi:10.3389/fneur.2025.1700753)
Supplement: Supplementary file 1 [file Table_1.docx]

Table S1. Multivariable logistic regression analysis for hemorrhagic transformation.

|  |  | 95%CI | |  |
| --- | --- | --- | --- | --- |
|  | OR | Lower limit | Upper limit | *P* value |
| Male | 0.281 | 0.281 | 2.629 | 0.790 |
| Age | 1.011 | 0.967 | 1.057 | 0.637 |
| Hypertension | 1.306 | 0.403 | 4.226 | 0.656 |
| Diabetes | 1.093 | 0.302 | 3.959 | 0.892 |
| Somking | 0.395 | 0.073 | 2.131 | 0.280 |
| NIHSS | 1.120 | 1.038 | 1.209 | 0.003 |
| ICAS | 2.113 | 0.278 | 6.091 | 0.470 |
| ECAS | 2.792 | 0.632 | 6.350 | 0.330 |
| IEAS | 1.156 | 0.884 | 1.512 | 0.290 |
| NIHSS: National Institutes of Health Stroke Scale; ICAS: Intracranial atherosclerotic stenosis; ECAS: Extracranial atherosclerotic stenosis; IEAS: Intracranial and extracranial artery stenosis. | | | | |

Table S2. Multivariable logistic regression analysis for 90-day poor clinical outcome.

|  |  | 95%CI | |  |
| --- | --- | --- | --- | --- |
|  | OR | Lower limit | Upper limit | *P* value |
| Male | 1.151 | 0.998 | 1.315 | 0.051 |
| Age | 1.036 | 1.001 | 1.073 | 0.044 |
| Hypertension | 0.545 | 0.198 | 3.503 | 0.841 |
| Diabetes | 1.191 | 0.362 | 3.915 | 0.773 |
| NIHSS | 1.198 | 1.105 | 1.298 | 0.001 |
| FBG | 0.952 | 0.822 | 1.102 | 0.507 |
| APTT | 1.050 | 0.966 | 1.141 | 0.255 |
| ICAS | 1.330 | 0.425 | 4.155 | 0.624 |
| ECAS | 1.218 | 0.343 | 4.319 | 0.760 |
| IEAS | 1.350 | 1.108 | 1.644 | 0.003 |
| NIHSS: National Institutes of Health Stroke Scale; FBG: Fasting Blood Glucose; APTT:Activated Partial Thromboplastin Time; ICAS: Intracranial atherosclerotic stenosis; ECAS: Extracranial atherosclerotic stenosis; IEAS: Intracranial and extracranial artery stenosis. | | | | |

Table S3. Multivariable logistic regression analysis for 90-day Death.

|  |  | 95%CI | |  |
| --- | --- | --- | --- | --- |
|  | OR | Lower limit | Upper limit | *P* value |
| Male | 3.642 | 0.900 | 4.685 | 0.064 |
| Age | 1.055 | 0.979 | 1.137 | 0.055 |
| Hypertension | 2.792 | 0.409 | 9.063 | 0.295 |
| Diabetes | 2.528 | 0.241 | 3.421 | 0.292 |
| NIHSS | 1.384 | 1.179 | 1.625 | 0.001 |
| PLT | 1.006 | 0.994 | 1.017 | 0.346 |
| FBG | 1.004 | 0.686 | 1.469 | 0.984 |
| ICAS | 0.818 | 0.060 | 3.169 | 0.880 |
| ECAS | 0.093 | 0.001 | 7.090 | 0.283 |
| IEAS | 1.047 | 0.915 | 2.362 | 0.111 |
| NIHSS: National Institutes of Health Stroke Scale; PLT: Platelet; FBG: Fasting Blood Glucose; ICAS: Intracranial atherosclerotic stenosis; ECAS: Extracranial atherosclerotic stenosis; IEAS: Intracranial and extracranial artery stenosis. | | | | |
